# Effectiveness of tactile and kinesthetic stimulation on weight and feeding patterns among Low birth weight neonates

**DOI:** 10.6026/9732063002001845

**Published:** 2024-12-31

**Authors:** Mahalakshmi B., Chauhan Roshaniben Rajendrakumar, Jamuna Rani P., Mahalakshmi N.B., Jothimani S., Siva Subramanian N., Ramani G.

**Affiliations:** 1 Department of Paediatric Nursing, Nootan College of Nursing, Sankalchand Patel University, Visnagar, Gujarat - 384315, India; 2 Department of Psychiatric Nursing, KMCH College of Nursing, Coimbatore, Tamil Nadu - 641048, India; 3 Department of Psychiatric Nursing, Shri Satyasai College of Nursing, Chenkgalpattu - 600127, Tamil Nadu, India; 4 Department of Psychiatric Nursing, Kongunadu College of Nursing, Coimbatore - 641109, India

**Keywords:** preterm neonates, low birth weight, tactile stimulation, kinesthetic stimulation, weight gain, feeding patterns

## Abstract

Preterm birth and low birth weight significantly contribute to neonatal morbidity, with affected infants often struggling with weight gain and feeding. This study investigated the effects of tactile and kinesthetic stimulation on these parameters in preterm and low birth weight neonates in Mehsana hospitals. Sixty neonates were split into an experimental group, receiving 15-minute tactile and kinesthetic sessions twice daily and a control group with standard care. Measurements on the first and seventh days showed that the experimental group had notable improvements, with weight gain increasing from 1.55 ± 1.30 to 3.50 ± 0.50 and feeding patterns similarly enhanced. In contrast, the control group's gains were modest and not statistically significant. Gestational age and birth weight were associated with positive responses to stimulation, especially in neonates with lower initial weights. These results indicate that tactile and kinesthetic stimulation could be an effective, low-cost method to support growth in vulnerable neonates.

## Background:

Preterm birth and low birth weight are significant global health issues, contributing to 15-20% of all births worldwide and accounting for approximately 20% of neonatal deaths each year [1]. According to the World Health Organization, an estimated 15 million babies are born preterm annually, with over 1 million of these neonates failing to survive due to complications related to their prematurity [2]. Infants born with a birth weight of less than 2500 grams or before 37 weeks of gestation often face various developmental challenges, including respiratory distress, feeding difficulties and neurodevelopmental delays, owing to their underdeveloped organs and immune systems. These challenges underscore the need for targeted interventions to support their growth and improve health outcomes during this critical period [3]. Preterm birth and low birth weight are major contributors to neonatal morbidity and mortality worldwide, posing significant health challenges for newborns and their families. These infants often face numerous developmental hurdles due to their underdeveloped organs and immune systems, leading to increased vulnerability to health complications. Consequently, there is a pressing need for interventions that support their growth and development during these critical early stages of life [4]. In recent years, tactile and kinesthetic stimulation have gained attention as non-invasive, cost-effective strategies for enhancing growth parameters such as weight gain and feeding efficiency in preterm and low birth weight neonates. Research has suggested that these forms of stimulation may positively influence neuromuscular development, immune function and physiological stability. Tactile stimulation involves gentle stroking and massaging of various body parts, while kinesthetic stimulation incorporates passive limb movements. Both techniques aim to mimic the physical interactions neonates would experience in a full-term intrauterine environment. This study explores the impact of tactile and kinesthetic stimulation on key developmental parameters, specifically weight gain and feeding patterns, in preterm and low birth weight neonates. Conducted in selected hospitals in Mehsana, this quasi-experimental study compares outcomes between neonates receiving the stimulation intervention and those in a control group without intervention. By assessing the effectiveness of these stimulation techniques, this research seeks to provide valuable insights that could inform neonatal care practices, ultimately aiming to enhance the health outcomes of this vulnerable population.

## Methodology:

## Research design:

A quasi-experimental, pre-test and post-test control group design was used [5] to assess the effectiveness of tactile and kinesthetic stimulation on selected developmental parameters among preterm and low birth weight neonates.

## Setting:

The study was conducted in two hospitals in Mehsana: Alka Multispecialty Hospital for the control group and Shri Sadguru Maternity and Nursing Home for the experimental group.

## Population and sample:

The target population consisted of preterm and low birth weight neonates admitted to the selected hospitals. A total of 60 neonates were recruited using a non-probability convenience sampling technique, with 30 neonates each assigned to the experimental and control groups.

## Variables:

[1] Independent variable: Tactile and kinesthetic stimulation.

[2] Dependent variables: Neonatal weight gain and feeding pattern.

## Intervention Procedure:

The experimental group received a combined intervention of tactile and kinesthetic stimulation:

[1] Tactile stimulation: Gentle stroking of the head, shoulders, back, arms and legs, with specific movements performed six times on each area.

[2] Kinesthetic stimulation: Passive movement of the arms and legs, with each limb moved six times.

This stimulation was administered one hour after feeding, twice daily, over a seven-day period. Each session included five minutes of tactile stimulation, followed by five minutes of kinesthetic stimulation, concluding with an additional five minutes of tactile stimulation.

## Data collection:

The study was conducted over six weeks, from June 15 to August 15, 2024. Permissions were obtained from the necessary authorities and ethical approval was secured from the institution's ethics committee. Oral consent was obtained from the guardians of all neonates. The primary parameters (weight and feeding pattern) were measured for each neonate in both groups on the first and seventh day of the intervention.

## Research tool:

Part I: Demographic details such as gestational age, birth weight, length, head circumference, chest circumference and birth order.

Part II: Grading system for weight and feeding patterns, categorizing scores as "Adequate," "Moderately Adequate," or "Inadequate."

## Data analysis:

Data were analyzed using SPSS version 23. Descriptive statistics were employed to describe demographic variables. Paired "t" tests compared pre-test and post-test scores within groups and independent "t" tests analyzed differences between groups. Additionally, chi-square tests examined associations between demographic variables and pretest parameter levels.

## Results & Discussion:

Table 1 shows Newborn characteristics including gestational age, birth weight, length, head circumference, chest circumference and birth order for experimental and control groups. Both groups showed similar distributions, ensuring comparability for evaluating the effects of tactile-kinesthetic stimulation. Table 2 showed that in experimental group significant improvements in weight gain (mean difference = 1.95, t = 8.724, p < 0.05) and feeding patterns (mean difference = 1.86, t = 6.95, p < 0.05). In contrast, the control group exhibited minimal, non-significant changes in weight gain (mean difference = 0.88, t = 5.416) and feeding patterns (mean difference = 0.65, t = 3.50), emphasizing the effectiveness of tactile-kinesthetic stimulation. Table 3 showed that gestational age, birth weight, length, head circumference and chest circumference were significantly related to pre-test scores in both groups (p < 0.05), while birth order was not significantly associated. This study demonstrates that tactile and kinesthetic stimulation significantly improves weight gain and feeding patterns among preterm and low birth weight neonates, with findings consistent across similar research. The study also explores associations between demographic factors (such as gestational age and birth weight) and growth outcomes, providing insights into how individual neonatal characteristics may interact with the effectiveness of tactile and kinesthetic interventions. In agreement with our results, Field *et al.* (1986) observed a substantial daily weight gain increase of 47% among preterm infants receiving tactile-kinesthetic stimulation, along with enhanced behavioral organization and shorter hospital stays [6]. This study suggests that such interventions may especially benefit neonates with lower initial weights and greater prematurity, as these infants often display more notable improvements with stimulation interventions. Dos *et al.* (2020) also found that tactile-kinesthetic stimulation reduced feeding intolerance and boosted weight gain, supporting the hypothesis that infants with lower gestational ages and birth weights may be more responsive to stimulation due to higher initial vulnerabilities [7]. Mathai *et al.* (2001) further supported the impact of tactile-kinesthetic stimulation on growth and neurobehavioral development, finding stronger benefits among very low birth weight neonates. Their findings suggest that, due to their developmental immaturity, these infants derive greater benefits from added stimulation, highlighting a positive association between lower birth weights and improved growth outcomes with tactile interventions [8].

Our study aligns with existing research demonstrating the benefits of tactile-kinesthetic stimulation (TKS) for low birth weight neonates. Similar to Aliabadi *et al.* (2013), who reported improved motor behavior and state regulation [9] and Elmoneim *et al.* (2023), who found significant improvements in weight, feeding patterns and respiratory stability, our findings highlight the positive impact of TKS on weight gain and feeding patterns [10]. Consistent with Field *et al.* (1986), our research further supports TKS as a cost-effective, non-invasive intervention that enhances growth and developmental outcomes in vulnerable neonates [11].

In line with these findings, Elmoneim (2023) observed that tactile/kinesthetic stimulation significantly improved weight, feeding patterns and respiratory stability in low birth weight neonates and associations with birth weight and gestational age influenced the effectiveness of these intervention [12]. Their study reinforces that younger, lower birth weight neonates often experience more substantial growth and developmental gains, potentially due to the heightened need for sensory stimulation that mimics the womb environment. The association analysis showed that all demographic variables, except for birth order, was significantly related to weight gain and feeding patterns in both the experimental and control groups. This finding aligns with previous studies, such as those by Zhang *et al.* (2023) and Janssen *et al.* (2007), which highlighted the importance of factors like gestational age, birth weight and physical measurements (*e.g.*, length and head circumference) in predicting neonatal growth outcomes [13-14]. The lack of significance for birth order suggests that it may not influence growth and feeding patterns as directly as biological factors tied to physical development. Similar to Karim *et al.* (2011), which also found birth order to be a non-significant factor, this study supports the view that physiological factors outweigh familial birth order in shaping neonatal growth trajectories [15].

## Figures and Tables

**Table 1:** Demographic characteristics of the participants (control and experimental groups)

**Demographic variable**	**Category**	**Experimental group (n=30)**	**Control group (n=30)**
Gestational Age	Less than 33 weeks	6 (20%)	6 (20%)
	33-34 weeks	11 (36.67%)	17 (56.67%)
	35-37 weeks	11 (36.67%)	6 (20%)
	Above 37 weeks	2 (6.67%)	1 (3.33%)
Birth Weight	Less than 1500 grams	4 (13.33%)	4 (13.33%)
	1500-1900 grams	17 (56.67%)	19 (63.33%)
	2000-2500 grams	7 (23.33%)	7 (23.33%)
	Above 2500 grams	2 (6.67%)	0 (0%)
Length	40-42 cm	9 (30%)	9 (30%)
	43-45 cm	15 (50%)	18 (60%)
	46-48 cm	6 (20%)	3 (10%)
Head Circumference	28-30 cm	8 (26.67%)	6 (20%)
	31-33 cm	16 (53.33%)	17 (56.67%)
	34-36 cm	6 (20%)	7 (23.33%)
Chest Circumference	25-27 cm	4 (13.33%)	5 (16.67%)
	28-30 cm	15 (50%)	18 (60%)
	30.5-33 cm	11 (36.67%)	7 (23.33%)
Birth Order	First	17 (56.67%)	15 (50%)
	Second	10 (33.33%)	11 (36.67%)
	Third	3 (10%)	4 (13.33%)

**Table 2:** Comparison of mean scores for weight gain and feeding pattern (experimental vs. control group)

**Group**	**Parameter**	**Mean Pre-Test Score**	**Mean Post-Test Score**	**Mean Difference**	**T-value**	**Significance**
Experimental Group	Weight Gain	1.55 ± 1.30	3.50 ± 0.50	1.95	8.724	Significant
	Feeding Pattern	1.60 ± 1.35	3.46 ± 0.57	1.86	6.95	Significant
Control Group	Weight Gain	1.50 ± 0.50	2.38 ± 1.14	0.88	5.416	Not Significant
	Feeding Pattern	1.55 ± 0.50	2.20 ± 1.35	0.65	3.5	Not Significant

**Table 3:** Association between pretest level of selected parameter scores with selected demographic variables with the preterm and low birth weight neonates in experimental group & control group.

**S.no**	**Demographic variables**	**Chi-square value (experimental)**	**Chi-square value (control)**
1	Gestational Age	13.783*	14.215*
2	Birth Weight	10.702*	9.642*
3	Length	11.481*	10.823*
4	Head Circumference	12.353*	11.658*
5	Chest Circumference	11.481*	12.114*
6	Birth Order	0.713 NS	1.012NS
Significance level: P < 0.05
NS = non-significant
*= significant
